# Noise causes cardiovascular disease: it’s time to act

**DOI:** 10.1038/s41370-024-00732-4

**Published:** 2024-12-10

**Authors:** Thomas Münzel, Andreas Daiber, Nicole Engelmann, Martin Röösli, Marin Kuntic, Jamie L. Banks

**Affiliations:** 1 Department of Cardiology, Cardiology I, University Medical Center of the Johannes Gutenberg-University, Mainz, Germany; 2 German Center for Cardiovascular Research (DZHK), Partnersite Rhine-Main, Mainz, Germany; 3 Center for Thrombosis and Hemostasis, University Medical Center of the Johannes Gutenberg-University, Mainz, Germany; 4 Department Epidemiology and Public Health, Swiss Tropical and Public Health Institute, Allschwil, Switzerland; 5 University of Basel, Basel, Switzerland; 6 Quiet Communities Inc., Concord, MA USA

**Keywords:** Epidemiology, Exposure assessment, Health studies, Human well-being, Meta-analysis, Noise pollution

## Abstract

**Background:**

Chronic transportation noise is an environmental stressor affecting a substantial portion of the population. The World Health Organization (WHO) and various studies have established associations between transportation noise and cardiovascular disease (CVD), such as myocardial infarction, stroke, heart failure, and arrhythmia. The WHO Environmental Noise Guidelines and recent reviews confirm a heightened risk of cardiovascular incidents with increasing transportation noise levels.

**Objective:**

We present a narrative review of the evidence from epidemiologic studies and translation studies on the adverse cardiovascular effects of transportation noise.

**Methods:**

We describe the results of a recent Umbrella+ review that combines the evidence used in the 2018 WHO Environmental Noise Guidelines with more recent (post-2015) high-quality systematic reviews of original studies. High-quality systematic reviews were included based on the quality of literature search, risk of bias assessment, and meta-analysis methodology using AMSTAR 2.

**Results:**

Epidemiologic studies show that exposure to high levels of road traffic noise for several years lead to numerous adverse health outcomes, including premature deaths, ischemic heart disease (IHD), chronic sleep disturbances, and increased annoyance. Mechanistically, noise exposure triggers oxidative stress, inflammation, endothelial dysfunction, and circadian rhythm disruptions. These processes involve the activation of NADPH oxidase, mitochondrial dysfunction, and nitric oxide synthase uncoupling, leading to vascular and cardiac damage. Studies indicate that chronic noise exposure does not result in habituation, and susceptible individuals, such as those with pre-existing CVD, are particularly vulnerable.

## Introduction

Extensive research has established the adverse health impacts of environmental exposures contributing to the exposome, specifically air pollution, on cardiovascular disease (CVD), including conditions such as myocardial infarction (MI), heart failure, arrhythmia, hypertension, and stroke [1]. Recent studies have highlighted particulate matter with a diameter of ≤2.5 µm (PM2.5) as a major air pollutant, contributing to ~7.9 million annual deaths [2]. Various studies have observed that proximity to major roads increased cardiovascular health problems such as ischemic heart disease (IHD) or hypertension [3, 4]. These studies cannot clarify whether the observed adverse effects are from air pollution or from noise.

Surprisingly, much less attention has been given to transportation noise despite urban and suburban areas experiencing high levels of both air pollution and noise. Noise, defined as “unwanted and/or harmful sound,” comes from transportation, occupational, leisure, residential, and industrial sources. With the present brief review, we want to focus on cardiovascular and metabolic health effects of transportation noise.

## Traffic noise exposure and the burden of disease

In 2020, the European Environment Agency (EEA) reported that many people remain exposed to high road traffic noise levels, estimating that at least 20% of the EU population lives in areas where transportation noise exceeds 55 dB L_den_ (reviewed in refs. [5, 6]). The World Health Organization (WHO) indicates adverse health impacts are likely at these noise levels, particularly at night when noise should not exceed 45 dB(A) (Table 1). The U.S. Department of Transportation estimated that in 2018 7.3% of the U.S. population was exposed to road traffic noise levels above 50 dB L_aeq,24_ (corresponding to a L_den_ of ≈53 dB), a number that the authors of the present review and even the U.S. Department of Transportation consider an underestimation of the real exposure levels of the U.S. population. In the EU, environmental noise, mainly from road transportation, is estimated to cause 12,000 premature deaths, 48,000 new cases of IHD, 6.5 million people experiencing chronic sleep disturbances, and 22 million individuals enduring significant annoyance annually. In 2020, it was estimated that 7.8 million, 5.2 million, and 7.9 million people in the U.S. were highly annoyed by aircraft, road, and rail traffic, respectively [7]. Urban expansion and increasing mobility demand are expected to raise the number of individuals exposed to road and railway noise by 2030, while aircraft noise exposure remains unchanged.

**Table 1:** WHO recommended limits for noise exposure levels and national legal thresholds for average noise exposure (https://www.euro.who.int/__data/assets/pdf_file/0009/383922/noise-guidelines-exec-sum-eng.pdf)^a^.

Noise source	L_den_	L_night_	Quality of evidence	EU/US threshold
Road noise	<53 dB	<45 dB	strong	No legally binding limits for ambient noise. Legal limits for L_REL,8h_ of 85–90 dB (US) and L_EX_ of 80–85 dB (EU) for occupational noise sources; peak (impulse) noise limits 135–140 dB.
Railway noise	<54 dB	<44 dB	strong	
Aircraft noise	<45 dB	<40 dB	strong	
Wind turbines	<45 dB	–	limited	
Leisure ambient noise	<70 dB (L_Aeq_,24 h)	–	limited	

^a^dB, decibel; L_den_, average sound pressure level over 24 h adjusted for day-evening-night with a penalty of 5 dB for the evening time (7–11 pm or 6–10 pm) and a penalty of 10 dB for the night time (11pm–7am or 10pm–6am); L_night_, average sound pressure level for night time (11pm–7am or 10pm–6am); L_Aeq_, average sound pressure level over 24 h (A-weighted means adjusted for the human acoustic range). The recommended limits are related to the most seriously exposed face of the building. Strong quality of evidence requires fast action of policy makers, whereas limited quality of evidence requires substantial discussions among the decision makers, also considering the opinion of scientists, clinicians and health care system representatives. L_REL,8h_, recommended exposure level over 8 h at workplace by the US CDC-associated National Institute for Occupational Safety and Health (NIOSH). L_EX_, recommended exposure level over 24 h or 7 d at workplace by the European agency for occupational safety and health (EU-OSHA). Reused with permission [46].

## Transportation noise and cardiovascular disease and death

Recent evidence highlights the impact of environmental noise on cardiovascular health [5, 6]. The WHO Environmental Noise Guidelines for the European Region (WHO ENG) included studies up to 2015 [8]. A recent Umbrella+ review identified subsequent studies, combining the newest high-quality systematic reviews with original studies post-2015 [9]. High-quality systematic reviews were included based on the quality of literature search, risk of bias assessment, and meta-analysis methodology using AMSTAR 2 [10]. Eligible original studies were required to use reliable noise exposure assessment methods and account for relevant confounders. For mortality and incident CVDs, only cohort studies were included, whereas prevalent hypertension studies also considered case-control and cross-sectional studies if they were population-based, large, and methodologically sound [9].

### Cardiovascular mortality

**Fig. 1:**
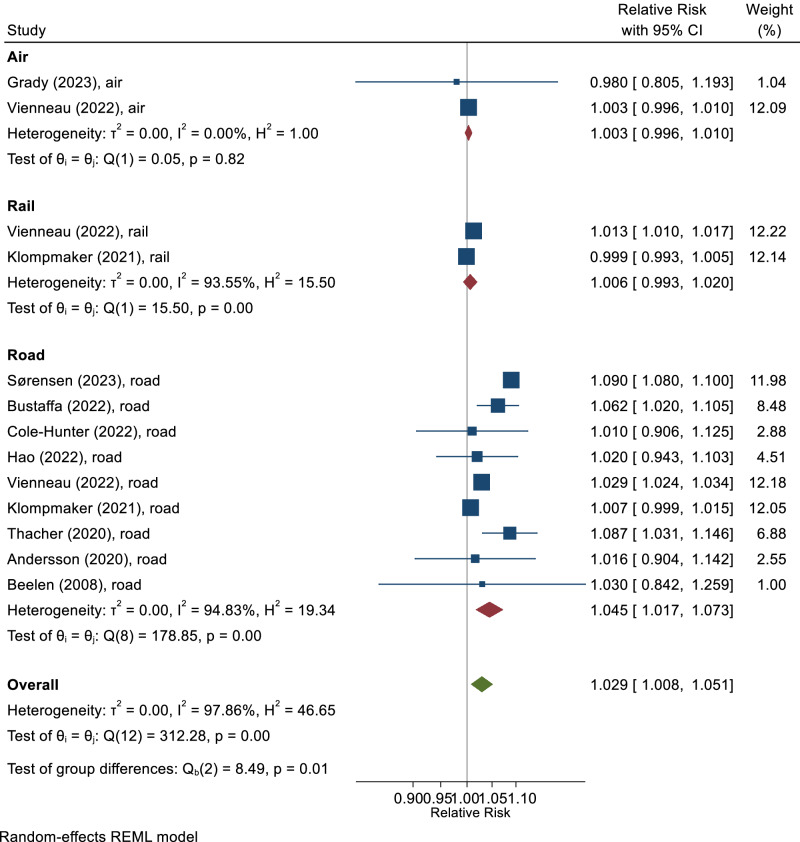
Meta-analysis of cohort studies on cardiovascular mortality in relation to transportation noise, stratified by source. Relative risks refer to a 10 dB increase in L_den_. Adapted with permission from [9].

The Umbrella+ review identified 61 cardiovascular (ICD-10: I00-I99) and IHD (I20-I25) mortality papers, out of which 12 prospective cohort studies on road, railway, and/or aircraft noise were eligible for meta-analysis. The pooled effect estimate for cardiovascular mortality per 10 dB(A) of road traffic noise was 1.05 (95% CI: 1.02–1.07) [5] based on nine studies (1.05 (95% CI: 1.03–1.08) for ischemic heart disease mortality). Only two studies each were available for railway and aircraft noise, both finding minimal effects on cardiovascular mortality. Figure 1 shows the meta-analysis results. A Swiss case-crossover study found that short-term exposure to aircraft noise was associated with CVD mortality, particularly exposure to nighttime aircraft noise of 40–50 dB(A) and >50 dB(A) within two hours prior to a CVD death [11].

### Ischemic heart disease (IHD)

The WHO ENG review found a relative risk (RR) of 1.08 (95% CI: 1.01–1.15) per 10 dB increase in L_den_ for IHD incidence due to road traffic noise [8, 12]. Recent studies, including a pooled Danish and Swedish cohort analysis [13] and a nationwide study from Denmark with over 2.5 million participants [14], reported hazard ratios (HRs) of 1.03 (95% CI: 1.00–1.05) and 1.05 (95% CI: 1.04–1.06) for road traffic noise. A meta-analysis combining WHO ENG [8, 12] with new studies found an RR of 1.04 (95% CI: 1.02–1.06) per 10 dB(A) increase in road traffic noise for IHD incidence [9].

### Stroke

The WHO ENG included one cohort study on road traffic noise and incident stroke, finding an HR of 1.14 (95% CI: 1.03–1.25) [8]. Nine subsequent studies mostly indicated positive associations with road traffic noise, with risks near unity for rail and aircraft noise. A pooled Danish and Swedish cohort analysis found an HR of 1.06 (95% CI: 1.03–1.08) per 10 dB(A) increase in road traffic noise [15]. The Umbrella+ review reported an RR of 1.05 (95% CI: 1.01–1.08) per 10 dB(A) increase in road traffic noise for incident stroke [9].

### Heart failure

The WHO ENG [8] did not address the effects of noise on heart failure. A 2023 meta-analysis found road traffic noise was associated with a 5% higher risk of heart failure per 10 dB(A) [16]. An updated meta-analysis found an RR of 1.04 (95% CI: 1.02–1.07) per 10 dB(A) increase in road traffic noise for heart failure [9].

### Arrhythmia and/or atrial fibrillation

Few reviews and cohort studies exist on noise and arrhythmia and/or atrial fibrillation. A Danish nationwide cohort study with over 3.5 million participants reported weak associations between atrial fibrillation and road, railway, and aircraft noise [17]. An updated meta-analysis found an RR of 1.01 (95% CI: 1.00–1.01) per 10 dB(A) increase in road traffic noise [9]. After the publication of the Umbrella review, a pooled analysis of eleven prospective Nordic cohorts found a RR of 1.02 (95% CI: 1.00–1.04) per 10-dB of 5-year mean time-weighted exposure, which changed to 1.03 (1.01–1.06) when implementing a 53-dB cut-off [18]. It should be mentioned that we did not identify literature on associations of transportation noise with sudden death, out-of-hospital cardiac arrest, or ventricular arrhythmias.

### Conclusions

The Umbrella+ review confirms associations between road traffic noise and various CVD diagnosis groups. Combining pooled effect estimates of IHD, stroke, hypertension, arrhythmia and heart failure results in a global CV risk increase of 3.2% (95% CI: 1.1–5.2%) per 10 dB higher road traffic noise (L_den_) [9]. Evidence is less pronounced for railway and aircraft noise, as road traffic noise is more prevalent, many people exposed to moderate levels of railway and aircraft noise may not hear it because the road traffic noise is substantially higher. As a consequence, road traffic noise is potentially masking the effects of railway and aircraft noise in source-specific analysis. Intervention studies are needed to demonstrate risk reduction after noise mitigation. It would be also important to design studies to include measures of traffic related pollutants (NO_2_ and PM2.5) and proximity of residence to roadway as complements to noise.

## Lower effect threshold of noise

The lower effect threshold of noise, below which no health effects are expected, is undetermined and likely varies by noise source and the different characteristics of noise (e.g., tonality, frequency). Different noise recommendations exist worldwide, with the EU using a 55 dB L_den_ threshold [8, 12] and the WHO recommending 53 dB(A) for road traffic noise [19]. Recent large cohort studies suggest effects starting around 45 dB L_den_ for various cardiovascular diagnoses and diabetes [9].

## Transportation noise and diabetes and obesity

Recent cohort studies have linked transportation noise, especially from road traffic, with a higher risk of diabetes (reviewed in ref. [5]). A meta-analysis found that a 10 dB(A) increase in road traffic noise was associated with a relative risk (RR) for diabetes of 1.06 (95% CI: 1.03–1.09). Studies have suggested that noise may affect sleep quality and contribute to metabolic changes leading to diabetes. Research also indicates that noise exposure may contribute to obesity (reviewed in ref. [5]). Several studies found associations between road traffic noise and increased measures of adiposity, suggesting that noise can affect weight gain throughout life.

## Noise and epigenetic changes: adverse impacts on the immune system and vascular function

Cross-sectional cohort studies have found that exposure to transportation noise can have an impact on the immune system. Two studies observed that noise increases levels of IL-12 and high-sensitivity CRP (C-reactive protein) while decreasing natural killer cell populations and activity, although the extent of noise effects on the immune system is not consistently uniform across all studies [20–22]. Furthermore, alterations in the immune system have been linked to elevated circulating cortisol levels and heightened noise sensitivity [21, 22]. Higher cortisol levels may also be related to nocturnal noise exposure and impaired circadian rhythm [23, 24].

Interestingly, a study based on the Swiss SAPALDIA cohort showed that long-term exposure to transportation noise and air pollution led to distinct and shared DNA methylation patterns, with enrichments in pathways related to inflammation (e.g., CRP), cellular development, and immune responses [25]. Findings in the same cohort suggested that chronic exposure to nocturnal intermittent train or road traffic noise increases arterial stiffness (reflecting endothelial dysfunction), as determined by pulse wave velocity [26]. This finding is supported by a German cohort study, showing that long-term exposure to nighttime road traffic noise is associated with subclinical atherosclerosis, especially in participants with early arterial calcification [27, 28].

In summary, these findings offer pathophysiological and molecular evidence from human studies, highlighting the effects of transportation noise on incident CVD. Notably, the results from these human studies, including stress pathways, inflammation, oxidative stress, arterial stiffness, and endothelial/cardiac dysfunction, align with mechanistic data from animal studies (reviewed in refs. [5, 6]).

## Noise annoyance

Noise annoyance, a psychological response to unwanted sounds, can be an early indicator of more severe health risks. It involves cognitive, emotional, and behavioral reactions influenced by personal, social, and situational factors. Recent studies have shown that transportation noise, particularly from roads and aircraft, is associated with increased annoyance levels, contributing to stress and negatively impacting cardiovascular health (reviewed in refs. [5, 6]). Annoyance from noise can lead to increased stress hormone levels and inflammation, further contributing to cardiovascular risk.

## Noise and amygdalar activation

**Fig. 2:**
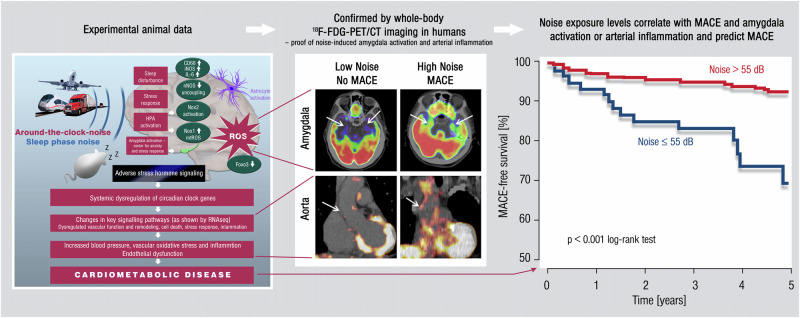
Key data on health effects of noise through the brain-heart/vessel-axis. *Left panel:* Main results of animal studies regarding brain-heart/vessel interaction. *Middle and right panel:* Proof-of-concept translational study in humans demonstrating the association between transport (road and aircraft) noise-induced cerebral (amygdala relative to cortical) metabolic activity and arterial inflammation increasing major adverse cardiovascular events (MACE) [29, 30]. Reused with permission from ref. [30].

The link between noise exposure and major adverse cardiovascular events (MACE) was observed in a 2020 study where stress-associated neural activity was associated with arterial inflammation in 498 healthy subjects without active cancer or CVD [29]. The neural activity was determined as the ratio of amygdala to regulatory cortical metabolic activity envisaged by the ^18^F-fluorodeoxyglucose positron emission tomography-computed tomography (PET–CT) imaging. At the same time, aortic inflammation was also determined using PET-CT to observe ^18^F-fluorodeoxyglucose uptake. The results indicated that the increased noise exposure level at the individual’s home address was linked to elevated amygdala activity, arterial inflammation, and higher risk of MACE, independently of confounders such as air pollution, socioeconomic status, and other established CVD risk factors. The study’s authors conclude that the association between higher noise exposure and MACE occurred via elevated amygdala activity and arterial inflammation (Fig. 2) [29, 30]. Interestingly, a similar pathway was previously observed to be responsible for the association between perceived stress and socioeconomic disparities (e.g., lower education or income) and CVD [31].

## Mechanistic insights: translational studies in humans and animals

### Oxidative stress and endothelial dysfunction

**Fig. 3:**
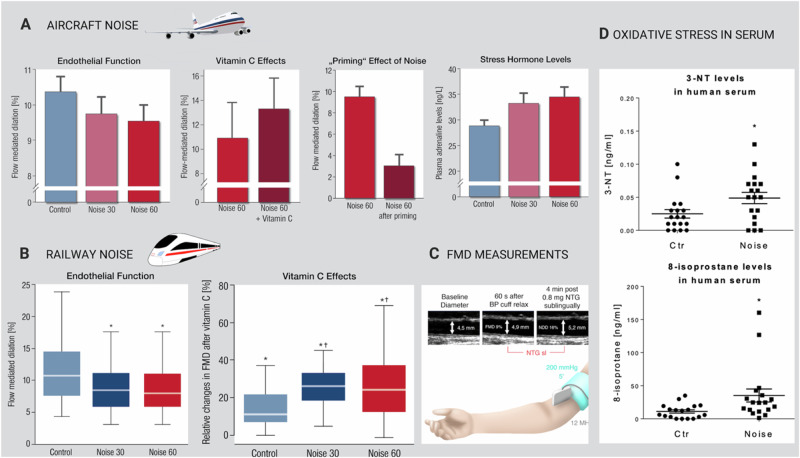
Key effects of noise observed in human field studies. **A** Effects of 30 and 60 aircraft noise events on the brachial artery (Noise30 and Noise60) of 70 healthy subjects. Vitamin C effects were assessed in a subgroup of the cohort. A priming effect of aircraft noise on endothelial function was observed, i.e., previous exposure to Noise30 caused Noise60 to have a significantly stronger reduction of flow-mediated dilation [52]. Serum adrenaline levels also increased significantly. **B** Effects of 30 and 60 railway noise events on flow-mediated brachial artery dilation in 70 healthy subjects. Vitamin C effects were assessed in a subgroup. **C** Methodology of FMD. **D** Effects of aircraft noise on oxidative stress markers (3-nitrotyrosine [3-NT] and 8-isoprostane) in serum that were measured in the samples of the aircraft noise study and published in ref. [32]. Adapted from [32] with permission. Copyright ©2018, Oxford University Press.

Translational studies in humans with and without CVD have demonstrated that exposure to transportation noise for one night (47 dB(A) L_eq_) leads to a significant increase in oxidative stress markers such as 3-nitrotyrosine and 8-isoprostane in serum and a significant degree of endothelial dysfunction as indicated by a reduction of flow-mediated dilation (FMD) (Fig. 3) (reviewed in refs. [5, 6]). Importantly, the deterioration of FMD was stronger in subjects with already established CVD. The acute administration of the antioxidant vitamin C has been shown to improve endothelial function, indicating the roll of oxidative stress in noise-induced vascular damage.

**Fig. 4:**
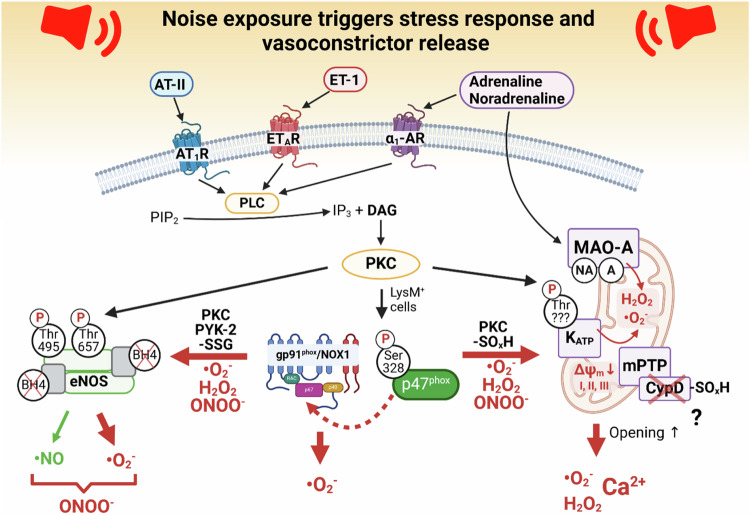
Oxidative stress pathways activated by noise. Noise causes stress hormone release (catecholamines and cortisol) and downstream endocrinal activation of vasoconstrictors activating common disease pathways, such as oxidative stress. Angiotensin II (AT-II) and endothelin-1 (ET-1) lead to the formation of diacylglycerol (DAG) from phosphatidylinositol 4,5-bisphosphate (PIP_2_), a potent activator of protein kinase C (PKC), via their receptors and the activation of phospholipase C (PLC). (1) PKC via phosphorylation of p47^phox^ at serine 328 causes activation of the phagocytic NADPH oxidase (NOX-2) and potentially NOX-1. The expression of NOX-2 is upregulated by noise-triggered immune cell infiltration (lysozyme M-positive (LysM^+^) cells) and systemic inflammatory conditions. NOX-2 (and NOX-1, especially in the brain) produces superoxide (O_2_^•−^) and via dismutation also hydrogen peroxide (H_2_O_2_). NOX-4 was not changed by noise and NOX-5 (relevant for humans) was not studied so far. (2) Dysfunction of endothelial nitric oxide synthase (eNOS) is mediated by noise-dependent activation of PKC and phosphorylation of threonine 495. Alternatively, NOX-2-dependent ROS formation may activate PKC [53] and protein tyrosine kinase 2 (PYK-2) [54, 55], causing adverse phosphorylation at tyrosine 657 and threonine 495. Uncoupling of eNOS may be induced by noise-driven oxidative depletion of tetrahydrobiopterin (BH_4_) and S-glutathionylation (-SSG) of eNOS by ROS originating from NOX-2 [56]. Semi-uncoupled eNOS may represent a potent source of peroxynitrite. (3) Noise also leads to mitochondrial ROS formation, generating both O_2_^•−^ and H_2_O_2_. Noradrenaline (NA) and adrenaline (A) originating from sympathetic activation are substrates of monoamine oxidases (MAO) that produce H_2_O_2_. NA and A can also activate PKC through adrenergic receptor (α_1_-AR). PKC seems to activate the mitochondrial K_ATP_ channel by phosphorylation of a threonine residue with subsequent depolarization of the mitochondrial membrane (ΔΨ_m_↓) and O_2_^•−^ formation from respiratory complexes I, II and III. Mitochondrial H_2_O_2_ / O_2_^•−^ and calcium are released to the cytosol upon the mitochondrial permeability transition pore (mPTP) opening (e.g., by thiol oxidation of the regulatory subunit cyclophilin D (CypD) [57]). K_ATP_ channel activation and mPTP opening can also be stimulated by redox-crosstalk with H_2_O_2_ (probably also O_2_^•−^ via peroxynitrite) derived from NOX-2 [58]. So far, there is no evidence for the role of xanthine oxidase in noise’s non-auditory (indirect) effects. This scheme was adapted from [35] with permission and created using biorender.com.

Preclinical studies revealed that oxidative stress in noise-exposed mice (72 dB(A) L_eq_, around-the-clock for 4 days) is primarily driven by the activation of NADPH oxidase (NOX-2), a key enzyme in inflammatory cells like leukocytes and macrophages that produces reactive oxygen species (ROS) but is also driven by a dysfunctional, uncoupled endothelial nitric oxide synthase [32]. Noise exposure also activates inflammatory pathways. It triggers the hypothalamic-pituitary-adrenal axis and the sympathetic nervous system, leading to the release of stress hormones like cortisol and catecholamines (Fig. 4) (reviewed in refs. [5, 6]). These hormones induce a pro-inflammatory state characterized by elevated levels of interleukins (IL-6, IL-1β) and proinflammatory monocytes. This inflammation can lead to vascular changes that contribute to the progression of atherosclerosis and other cardiovascular conditions.

Recently we also demonstrated that noise exposure can dysregulate gene networks within the vasculature. This includes the upregulation of genes involved in TGF-beta signaling, autophagy, and growth regulation and downregulation of genes associated with cell cycle control and apoptosis [33]. These changes in gene expression further impair endothelial and vascular signaling, contributing to cardiovascular dysfunction.

An additional finding from our preclinical studies is that nighttime noise exposure has a more detrimental effect than daytime noise. We demonstrated that nighttime noise, as opposed to daytime noise, led to significantly higher blood pressure, a greater increase in neurohormonal release, elevated oxidative stress in vascular tissue, increased endothelin-1 expression within the vasculature and interestingly no endothelial dysfunction at all. These factors may explain, at least in part, why nighttime noise contributes to greater vascular stiffness and higher blood pressure compared to daytime noise (for review, see [34]). Moreover, we observed circadian clock dysregulation, primarily involving the downregulation of FOXO3, a transcription factor serving as a central signaling hub. We tested the effect of bepridil, a FOXO3 activator, calcium antagonist, anti-anginal, and class IV anti-arrhythmic drug. Bepridil prevented noise-induced endothelial dysfunction, increased FOXO3 mRNA expression, and reduced vascular and cerebral oxidative stress [32]. Based on these findings, we hypothesized that the adverse effects of nighttime noise are partly due to circadian rhythm disruption, as noise during sleep causes sleep fragmentation and reduced sleep quality, thereby amplifying stress responses leading to more pronounced oxidative stress and endothelial dysfunction [32–34]

This disruption affects central and peripheral circadian clocks, contributing to metabolic and cardiovascular dysfunction. Nighttime noise exposure causes also a significant downregulation and uncoupling of neuronal nitric oxide synthase (nNOS), leading to a neuroinflammatory phenotype [32] that probably affects cognitive functions and increases cardiovascular risk.

Noise exposure affects the neuroendocrine system by elevating levels of angiotensin II and endothelin-1, hormones that regulate blood pressure and fluid balance (Fig. 4). This elevation increases oxidative stress and inflammation in the brain’s microvasculature and conductance vessels, contributing to hypertension and other cardiovascular issues (reviewed in refs. [5, 6]). The sympathetic nervous system activation due to oxidative stress further releases catecholamines, which can exacerbate cardiovascular damage. The following sections briefly explain how stress hormone-mediated receptor signaling can activate sources of ROS (superoxide, O_2_^•−^ and hydrogen peroxide, H_2_O_2_) and lead to oxidative stress conditions (reviewed in ref. [35]).

### NADPH oxidases

NOX-2 (gp91phox), the phagocyte isoform of NADPH oxidases, is a key enzyme in host defense. Whereas other NADPH oxidase isoforms (e.g., NOX-1, NOX-4, NOX-3, and DUOX-2) play a role in noise-induced hearing loss, the role of NADPH oxidases in the non-auditory (indirect) pathology is less explored. Upon noise exposure, NOX-2 protein and NOX-2 mRNA levels are consistently upregulated in the murine aorta and heart [32, 33]. Also, a more pronounced activation state of NOX-2 was reported for noise-exposed mice, which was driven by angiotensin-II or endothelin-1 dependent diacylglycerol-mediated protein kinase C (PKC) activation with subsequent Ser328 phosphorylation of p47phox, the cytosolic regulator, and activation of NOX-2 (Fig. 4). Evidence of oxidative stress is readily detectable in the aorta, heart, and brains of mice exposed to noise [32, 33] and the serum of noise-exposed healthy subjects. Importantly, mice with a genetic deletion of NOX-2 gene or ablation of inflammatory monocytes (LysM^+^ cells) are protected from this oxidative stress and the subsequent endothelial dysfunction [32, 36]. Further support for a central role of NOX-2 in noise-mediated pathophysiology comes from studies showing an additive upregulation of NOX-2 protein in noise-exposed hypertensive and MI mice (reviewed in ref. [5]).

### Mitochondria

Mitochondria are well-known producers of ROS and are known to contribute to oxidative damage in IHD and hypertension (reviewed in ref. [35]). Different noise sources and patterns were reported to cause mitochondrial damage in the form of cardiac fibrosis, enlarged cardiac mitochondria, swelling, matrix dilution, cristolysis, DNA damage and reduced connexin 43 contents (reviewed in ref. [5]). These observations can be linked to high noradrenaline levels, monoamine oxidase (MAO) activity, disturbed mitophagy, potentially negatively impacting permeability transition (e.g., mPTP), and calcium handling. Catecholamines (or serotonin) serve as MAO substrates enabling significant ROS formation. Accordingly, an additive increase in mitochondrial superoxide levels was seen in the hearts of noise-exposed mice with MI in conjunction with impaired mitochondrial respiration and oxygen handling [37]. Pathways that could be involved in noise-dependent mitochondrial ROS formation are shown in Fig. 4.

### Uncoupled nitric oxide synthases

Due to the excessive superoxide formation in noise-exposed animals, endothelial NOS (eNOS) in the aorta (and neuronal - nNOS in the brain) uncouples, which means that it transforms into a source of O_2_^•−^ and H_2_O_2_ instead of proper synthesis of nitric oxide (^•^NO). NOS uncoupling was previously demonstrated in tissues of noise-exposed mice by dihydroethidium staining in the presence of the eNOS inhibitor N^G^-nitro-L-arginine methyl ester (L-NAME) [32, 33]. eNOS is redox-sensitive because of its reliance on a readily oxidizable cofactor, tetrahydrobiopterin (BH_4_). Without BH_4_, eNOS cannot produce ^•^NO, but instead produces O_2_^•−^. The concomitant formation of ^•^NO and O_2_^•−^ by uncoupled eNOS generates peroxynitrite, which in term reacts with proteins to result in their tyrosine nitration as observed in noise-exposed mice and humans [32, 33]. eNOS uncoupling diminishes ^•^NO bioavailability in the aortas of noise-exposed mice as determined by the direct quantification of ^•^NO using electron spin resonance spectroscopy. Adverse dysregulated phosphorylation by excessive ROS could further aggravate eNOS dysfunction. Another redox-dependent uncoupling mechanism is eNOS S-glutathionylation, which was also increased in the aorta and heart of noise-exposed mice [32, 33]. The latter effect was not observed in NOX-2–deficient mice and was aggravated in noise-exposed hypertensive mouse hearts. The noise-triggered adverse regulation of eNOS that switches the enzyme to a peroxynitrite and superoxide source is shown in Fig. 4.

## Lack of tolerance development to cardiovascular health effects of noise

Chronic exposure of mice to aircraft noise for 4 weeks does not result in habituation concerning the cardiovascular side effects. Persistent endothelial dysfunction and elevated blood pressure were observed in studies exposing animals to noise for up to 28 days [38]. The formation of ROS increased over time, particularly in the aorta, heart, and brain. This oxidative stress was marked by a peak oxidative burst in whole blood after 4–7 days. Additionally, increased superoxide in the brain was associated with the downregulation of neuronal nitric oxide synthase (NOS3) and FOXO3 genes. Inflammatory markers like VCAM-1 mRNA were consistently upregulated, indicating that mice did not acclimate to chronic noise stress, and endothelial dysfunction and inflammation persisted throughout the exposure period.

## Noise preconditioning and myocardial infarction

The impact of noise on susceptible patients, such as those with acute coronary syndromes [39], was also studied on a mechanistic basis by exposing mice to 72 dB(A) noise levels with peaks at 85 dB(A) for up to 4 days [37]. This exposure activated pro-inflammatory gene expression related to myeloid cell adhesion and diapedesis pathways. Noise exposure led to increased adhesion and infiltration of inflammatory myeloid cells in vascular and cardiac tissues, and a higher percentage of leukocytes showed a pro-inflammatory phenotype characterized by ROS and upregulation of NOX-2 and NF-κB phosphorylation. This resulted in “priming” of the heart for ischemic damage. Subsequent MI caused more pronounced endothelial dysfunction and elevated vascular ROS levels in noise-preconditioned animals (reviewed in ref. [5]).

Translational studies in the Gutenberg Health Study Cohort found that individuals with prior noise exposure and annoyance had elevated baseline CRP levels and a more significant decline in left ventricular ejection fraction after an MI [37]. People with acute coronary syndromes were particularly susceptible to aircraft noise, with a hazard ratio (HR) of 1.24 (95%-CI: 0.97–1.58) per 10 dB increase in L_den_ aircraft noise [39]. Combined analysis showed an HR of 1.31 (95%-CI: 1.03–1.66) for recurrence of cardiovascular events and all-cause mortality, indicating high susceptibility of CVD patients to noise.

## Recovery time for cardiovascular system after noise stress

Recovery from noise-induced endothelial dysfunction in the aorta was possible within 1–4 days of noise cessation in mice [40]. Acetylcholine-dependent relaxation measurements confirmed this recovery. Partial correction of vascular oxidative stress and blood pressure and normalization of inflammatory markers such as VCAM-1 and IL-6 were observed. However, endothelial dysfunction and inflammation in cerebral microvessels did not improve, suggesting that microcirculation requires longer recovery to reverse noise-induced vascular dysfunction (reviewed in ref. [5]).

## Modifying noise-induced health effects through α1AMPK activation

Non-pharmacological approaches like physical activity, a balanced diet, and weight management are effective in preventing and treating CVD and diabetes [41]. It is known that exercise can mitigate the impact of air pollution-induced CVD and mortality. Activation of α1AMPK through exercise, intermittent fasting, and pharmacological methods (e.g., AICAR) was explored in mice exposed to aircraft noise [42]. Noise exposure-impaired endothelial function in the aorta, mesenteric arteries, and retinal arterioles was accompanied by increased vascular oxidative stress and asymmetric dimethylarginine formation. α1AMPK activation effectively prevented endothelial dysfunction and oxidative stress, supported by RNA sequencing data. Absence of endothelium-specific α1AMPK worsened noise-induced vascular damage, nullifying the protective effects of exercise or fasting, highlighting the importance of α1AMPK activation in mitigating noise-induced cardiovascular damage (reviewed in ref. [5]).

## Prevention and mitigation strategies to reduce transportation noise

Transportation noise is a public health problem affecting large swaths of the global population. The onus is on policymakers and other decision makers to take action to protect the public from the harms of environmental noise. Particular attention should be paid to populations exposed to the highest levels of noise which, at least in the United States, tend to affect low income communities disproportionately [43, 44], as well as other vulnerable populations, such as those with pre-existing CVD. Professional societies, e.g., American Heart Association, American College of Cardiology, European Society of Cardiology, should incorporate environmental noise into CVD prevention guidelines and educational materials. Policymakers should incorporate environmental noise criteria into program and policy screening tools and enact measures to mitigate existing sources of harmful environmental noise. The US EPA has provided a cumulative risk assessment framework that could accommodate noise (https://www.epa.gov/risk/framework-cumulative-riskassessment). By applying the cumulative risk framework provided by the US EPA (Environmental Protection Agency) [45], a more holistic approach to policy development and mitigation is potentially achieved.

The mitigation measures proposed below were reviewed extensively in refs. [5, 46]. Local authorities can employ several strategies to mitigate road, railways, and aircraft noise as outlined in a policy brief of the European Commission [47]. Priority measures are at the source. Special noise-reducing asphalt can decrease noise by 3 to 6 dB(A). Reducing speed limits can decrease noise by ~1 dB(A) per 10 km/h reduction. Low speed (20 miles per hour) in combination with electrification of cars, reduces noise in urban areas to a large extent. At higher speed, electrification is less effective as the sound generated by the interaction of tires and pavement is the dominating noise source. Promoting the use of low-noise tires can potentially reduce noise by 2–3 dB(A). Investing in urban infrastructure such as bike lanes, ride-sharing programs, and public transportation can also help to reduce urban noise levels as well as air pollution levels. As an ultimate measure, sound proof windows reduce indoor noise substantially and for road and railway noise, erecting barriers along busy lines in densely populated areas can reduce noise levels by up to 10 dB(A).

To address aircraft noise, implementing GPS-guided routes can help avoid densely populated areas, thus reducing noise impact. Prohibiting take-offs and landings during nighttime hours can significantly reduce sleep disturbances. Continuous descent approaches with steeper descents, and lower throttle settings can minimize noise during landings. Furthermore, promoting the development and use of quieter aircraft technology can have a long-term impact on reducing noise pollution from aviation.

For railway noise, regular maintenance and grinding of tracks can help reduce noise generated by train operations. Replacing traditional cast-iron block brakes with composite materials can lower noise levels during braking. Prohibiting railway operations near residential areas during nighttime can help reduce disturbances. Investing in vibration-damping track systems and sound barriers along railway lines can also mitigate noise pollution from trains.

By combining these strategies, authorities can substantially reduce noise, particularly in densely populated areas. A comprehensive approach that includes technological advancements, infrastructure improvements, and policy changes will most effectively address transportation noise pollution and help to improve public health.

## Interaction between transportation noise and air pollution

Noise and air pollution often co-occur, having a common source in fossil fuel-powered vehicles, equipment, and machinery. Their combined exposure can adversely affect cardiovascular health, leading to cumulative risk increases in humans as reported for diabetes [48], stroke [49], and MI [50]. Animal studies using combined exposure systems revealed that both stressors independently cause endothelial dysfunction and oxidative stress, and their combination leads to more severe cardiovascular damage [51]. This interaction underscores the importance of considering multiple environmental factors in assessing cardiovascular risk.

## Conclusions

Transportation noise significantly impacts cardiovascular health through mechanisms involving oxidative stress, inflammation, endothelial dysfunction, gene dysregulation, circadian rhythm disruption, metabolic changes, and psychological stress. Translational studies in humans and experimental animals have provided valuable insights into these processes, emphasizing the need for comprehensive strategies to mitigate noise pollution and its health impacts. Based on the existing evidence concerning noise, it’s time now for immediate policy actions to decrease exposure to noise. While this is an action that is supported by the evidence, translation of science into policy and then the implementation of such policies through regulation, rules or guidance takes a long time. Thus we have to consider tactical approaches that can be implemented in the short-term together with health professionals, who can advise their patients to reduce exposure. Thus, we are recommending both a longer-term strategic approach as a solution and a shorter-term tactical approach.

Noise should be acknowledged as a significant cardiovascular risk factor along with other environmental hazards such as ambient air pollution and exposure to chemicals, e.g., in the CVD prevention guidelines of professional societies. Public officials and decision makers should act to reduce public exposure to harmful levels of noise and adhere to the national limits as well as WHO recommendations (Table 1). Further studies are needed to explore the interactions between noise and other environmental stressors and effective public protection interventions.
